# Antimicrobial Effects of Conditioned Medium From Amniotic Progenitor Cells *in vitro* and *in vivo*: Toward Tissue Regenerative Therapies for Bovine Mastitis

**DOI:** 10.3389/fvets.2019.00443

**Published:** 2019-12-19

**Authors:** Anna Lange-Consiglio, Claudia Gusmara, Emanuela Manfredi, Antonella Idda, Alessio Soggiu, Viviana Greco, Luigi Bonizzi, Fausto Cremonesi, Alfonso Zecconi

**Affiliations:** ^1^Department of Veterinary Medicine (DIMEVET), Università degli Studi di Milano, Milan, Italy; ^2^Reproduction Unit, Centro Clinico-Veterinario e Zootecnico-Sperimentale di Ateneo, Università degli Studi di Milano, Milan, Italy; ^3^Private Practitioner, Milan, Italy; ^4^Institute of Biochemistry and Clinical Biochemistry, Università del Sacro Cuore Roma, Rome, Italy; ^5^Fondazione Policlinico Universitario Agostino Gemelli IRCCS, Rome, Italy

**Keywords:** conditioned medium, mastitis, bacteria, regenerative medicine, bovine

## Abstract

There is increasing evidence to suggest that, in addition to their regenerative effect, mesenchymal stromal cells (MSCs), and their secretome have an anti-inflammatory and antimicrobial role in the innate immune response in conditions such as sepsis. However, there is no published information on the effect of MSCs in bovine mastitis. Mastitis often results in extensive tissue damage due to multi-microorganism co-infection. This study investigated the ability of amniotic-derived conditioned medium (CM), *in vitro* and *in vivo*, to counteract microbial action and restore healthy tissue capable of milk production. Following determination of a dose–response curve, 10,000 colony-forming units (CFU) of *Staphylococcus aureus* (*S. aureus*) were inoculated into bovine mammary epithelial cell culture with and without 10% CM (supplemented either at the time of bacteria inoculation or after 4 h). Acridine orange staining was used to assess cell viability/apoptosis. Additionally, an *in vivo* study was performed using 48 dairy cows with acute and chronic mastitis, treated with CM (treated group) or antibiotics (control group). *In vitro* results showed that CM can attenuate bacterial growth, as evaluated by the number of CFU. After 24 h of culture with *S. aureus*, 89.67% of mammary epithelial cells treated with CM were still alive, whereas all cells cultured without CM were dead. Rates of epithelial cell survival (60.67%) were similar when CM was added 4 h after bacteria inoculation. There was no difference in somatic cell count between cases of acute mastitis in the CM-treated or control group in the *in vivo* study. However, relapses in chronic mastitis were less common in the group receiving CM. Our results show that CM is able to mitigate bacterial growth *in vitro* and may be particularly useful in the treatment of chronic mastitis, aiding restoration of milk production in cows that would otherwise be removed from the production cycle.

## Introduction

Regenerative medicine relies on transplantation of mesenchymal stromal cells (MSCs) for the treatment of pathology in both human and veterinary medicine. However, engraftment of the transplanted MSCs has only been documented in a few cases (1–3). Indeed, only a small percentage of transplanted MSCs are able to engraft and survive in the inhospitable environment of a lesion (4–7). Recent data suggest that the action of transplanted MSCs might involve the secretion of mediators that activate the regenerative processes in the injured tissue. Conditioned medium (CM), secreted by cells during culture, has been demonstrated to promote the structural and functional regeneration of cardiac (6, 8), renal (5, 9), spinal cord (10), lung (11), and tendon (12) tissues. Lange-Consiglio et al. (12) reported the process of healing in tendons treated with CM to be comparable to that achieved by the MSCs of origin. This suggests that the beneficial properties of MSCs are attributable to paracrine stimulation of tissue-resident stem cells. These effects may be ascribed to bioactive factors known to suppress apoptosis and fibrosis, improve angiogenesis, induce mitosis, promote stem cell differentiation, and modulate the innate immune response (13). In addition to releasing soluble factors into the CM, MSCs also release non-soluble factors, such as extracellular microvesicles (MVs) containing microRNAs. All these components can be considered as essential intermediaries of cell-to-cell communication (14). Thus, the substances produced by MSCs may be the main mediators of their regenerative action. Pereira et al. (15) showed that CM secreted by human cord blood cells has a high concentration of soluble factors with proliferative, chemotactic, and immunomodulatory action. The components of CM included growth factors, cytokines, chemokines, amino acids (valine, threonine, alanine, methionine, and glutamine), glucose, and molecules with low molecular weight (lactate, acetate, and pyruvate). Rossi et al. (16) identified soluble factors that had an inhibitory action on the proliferation of T lymphocytes in CM derived from cells isolated from human amniotic membrane and suggested that prostaglandins might be responsible for this effect.

Although it is now recognized that the regenerative effects of MSCs are mediated by paracrine stimulation of growth factors secreted in CM, less is known about their antimicrobial action. Some preclinical data indicate that MSCs and their secretome have antimicrobial properties (17–19). Studies using models of infection-induced organ damage have shown that MSCs can exhibit antibacterial properties by the release of soluble molecules, such as the peptides catelicidine LL-37 (20) and lipocalin-2 (21), which lead to the clearance of bacteria. Park et al. (22) demonstrated that MVs released by MSCs had an antimicrobial effect in post-mortem perfused human lung with severe bacteria pneumonia.

Bovine mastitis is an inflammatory process of the mammary gland predominantly associated with a bacterial infection and it is usually treated with intramammary antibiotics. Its effects on mammary cell numbers and function result in altered mammary secretion. The severity of the infection determines whether tissue damage can be reversed with treatment, and milk production comparable to that prior to the infection be regained. Therefore, a therapeutic approach that is both antimicrobial and regenerative would be ideal.

In view of the above, one aim of our study was to test the effects of CM on epithelial mammary cells stressed by *Staphylococcus aureus* (*S. aureus*). A further aim was to conduct a preclinical study to evaluate the potential of the *in vivo* administration of CM for regenerative based therapy of acute and chronic bovine mastitis.

CM from amniotic cells was used. These cells represent an intermediate state between embryonic and adult cells (23–26). Cells from extra-fetal tissues possess a greater proliferative and differentiative potential and have longer telomeres than cells derived from adult tissues (27–29). Furthermore, they are able to prevent fetal rejection due to their low immunogenicity and their immunomodulatory characteristics (30). Secretomes of amniotic derived cells have been shown to attenuate *in vitro* lipopolysaccharide (LPS)-induced inflammation in equine endometrial and tendon cells and in macrophages (31–33). These characteristics represent a promising therapeutic option in many inflammatory diseases.

## Methods

Chemicals, cell culture media, and supplements were obtained from Sigma Aldrich, Milan, Italy, unless otherwise specified, and tissue culture dishes were purchased from Euroclone, Milan, Italy.

### *In vitro* Study

#### Amniotic Membrane Collection, Cell Isolation, and CM Production

Allanto-amniotic membranes were collected following standard veterinary practice and in accordance with 2010/63 EU directive on animal protection and Italian Law (D.L. No. 116/1992) as previously described by Corradetti et al. (34). Briefly: the allanto-amnion was kept at 4°C and processed within 12 h. The amniotic membrane was separated from its juxtaposed allantois and cut into small pieces (about 9 cm^2^ each) that were digested with 0.93 mg/ml collagenase type I and 20 mg/ml DNAse (Roche, Mannheim, Germany) for ~3 h at 38.5°C. Debris were removed using a 100-mm cell strainer and released cells were collected by centrifugation at 200×*g* for 10 min. Amniotic cells (AMCs) were cultured in a medium composed of HG-DMEM supplemented with 10% FBS, penicillin (100 UI/ ml)–streptomycin (100 mg/ml), 0.25 mg/ml amphotericin B, 2 mM L-glutamine, and 10 ng/ml epidermal growth factor.

CM was obtained by culturing AMCs at passage 3 in ultra-culture serum-free medium (Euroclone), without antibiotics, for 96 h without replacement of medium. The medium from each flask was collected, pooled, centrifuged at 700×*g* to remove cellular fragments, and kept at −80°C. This method was carried out for cells isolated from three placentas.

For the *in vitro* and the *in vivo* experiments, the CM was lyophilized and preserved at 4°C until use. Before use, the CM was resuspended in sterile water to one-quarter of the initial volume.

#### Epithelial Mammary Cell Culture

A conventional clonal cell line (BME-UV) from udder primary epithelial cells (35) was used. These cells are known to synthesize several milk components.

BME-UV cells were cultured in medium containing 40% Ham F-12 nutrient mixture, 30% Roswell Park Memorial Institute (RPMI) 1640 medium, 20% NCTC 135 (Gibco, Paisley, UK), 10% fetal bovine serum, 0.1% lactalbumin hydrolysate, 1.2 mM glutathione, 1 μg/ml hydrocortisone, 1 μg/ml insulin, 0.1% lactose, 10 μg/ml L-ascorbic acid, and 1% penicillin/streptomycin.

The cells were seeded onto 12-well plates at a density of 3 × 10^3^ cells/cm^2^ and cultured at 38.5°C with 5% CO_2_. All trials were performed on 80% of confluence.

#### *Staphylococcus aureus* and Dose–Response Curve

The *S. aureus* strain used in this *in vitro* study was isolated from a clinical mastitis case. This strain is resistant to beta-lactams. The spectrum of antibiotic sensitivity of the microorganism was assessed by the VITEK® 2 system using AST-GP79 test cards (BioMérieux, Marcy l'Étoile, France), following the manufacturer's instructions. Data on antibiotic sensitivity are represented in Table 1.

**Table 1:** Spectrum of the antibiotic sensitivity of the *Staphylococcus aureus* strain used for the *in vitro* assays.

**Antimicrobial**	**MIC**	**Interpretation**
Benzilpenicillin	≥0.5	R
Oxacillin	≤0.25	S
Cefalotin	≤2	S
Ceftiofur	≤0.5	S
Cefquinome	≤1	S
Amikacin	≤2	S
Gentamycin	≤0.5	S
Kanamycin	≤4	S
Neomycin	≤2	S
Enrofloxacin	≤0.5	S
Erythromycin	≥8	R
Tilmycosin	≥4	R
Tylosin	≥32	R
Clindamycin	≤0.12	S
Tetracycline	≤1	S
Florphenicol	≤4	S
Trimethoprim/Sulfametoxazol	≤10	S

*MIC, Minimal inhibitory concentration; R, resistant; S, sensible*.

The stress induced by *S. aureus* was evaluated by viability and apoptotic staining on a dose–response curve in three replicates. BME-UV cells were seeded at a density of 3 × 10^3^ on 12-well plates and *S. aureus* was inoculated at the rate of 100, 1,000, and 10,000 CFU/ml for 4–8, 12, and 24 h at 38.5°C in 5% of CO_2_. The concentration of CFU was assessed spectrophotometrically and by serial dilution in order to verify the accuracy of the inoculated quantities.

#### *Staphylococcus aureus* CM Treatment

BME-UV seeded at a density of 3 × 10^3^ on 12-well plates with 1 ml of culture medium were studied at different conditions: (1) with *S. aureus* at a final concentration of 10,000 CFU/ml (identified as the optimal concentration after dose–response curve analysis); (2) with 10% CM; (3) with *S. aureus* and CM; and (4) untreated (control group, CTR).

In condition 3 (*S. aureus* and CM), the CM was added either concurrently with the bacterium at time 0 or after 4 h to allow bacterial growth.

In all conditions, cell culture was performed in a CO_2_ incubator at 38.5°C for 24 h.

Each group was assessed at 4, 8, 12, and 24 h. At each time, supernatants and cells were collected to evaluate bacteria count and to perform viability and apoptotic staining.

#### Bacterial Count

At the end of each incubation time (4, 8, 12, and 24 h) and for each experimental condition, supernatants were transferred into sterile plastic tubes and 1 ml was used for bacteria counts. Bacteria counts were carried out by the standard dilution method.

The supernatant (1 ml) was diluted 10^−7^. For each dilution, 100 μl was inoculated on two plates of blood agar using the spread method. After incubation at 37°C overnight, the plate with bacterial colonies between 15 and 150 were examined and the colonies were counted. The data were expressed as CFU per ml.

#### Viability and Apoptotic Staining

Staining was performed to classify cells as viable, apoptotic, and necrotic as described in the literature (36). Staining was performed using 1 μl of a solution of acridine orange (5 mg in 1 ml of ethanol) combined with 1 μl of propidium iodide and 1 ml of phosphate buffer solution.

At each time point, the cells were detached with trypsin and centrifuged at 250×*g* for 10 min. Fifty microliters of pellet was diluted with 50 μl of dye. The analysis was carried out immediately under fluorescence light microscopy (Olympus BX51, Japan) at a magnification of 40×. Acridine orange dye was excited at 460 nm while the emission wavelength was set at 650 nm. Propidium iodide was excited at 535 nm while the emission wavelength was set at 617 nm.

#### Protein Identification in CM

Protein digestion was performed according to the filter-aided sample preparation (FASP) protocol that combines both protein purification and digestion (37, 38). Each biological sample was run in quadruplicate. Briefly, reduction (DTT 8 mM in urea buffer −8 M urea and 100 mM Tris), alkylation (IAA 50 mM in urea buffer −8 M urea and 100 mM Tris), and digestion by trypsin at a final concentration of 0.01 μg/μl (Promega Italia srl, Milan, Italy) were performed on filter tubes (Nanosep centrifugal device with Omega membrane-30 K MWCO, Sigma-Aldrich). LC-MS analysis was performed as previously described (39). First, 500 fmol/μl of digestion of enolase from *Saccharomyces cerevisiae* (P00924) was added to each sample as an internal standard, tryptic peptides were separated, and then 0.25 μg of each digested sample was loaded onto a Symmetry C18 5 μm, 180 μm × 20 mm precolumn (Waters Corp., Milford, MA, United States) and subsequently separated by a 90-min reversed-phase gradient at 300 nl/min (linear gradient, 2–85% CH3CN over 90 min) using a HSS T3 C18 1.8 μm, 75 μm × 150 mm nanoscale LC column (Waters Corp.) maintained at 40°C. The separated peptides were analyzed on a high-definition Synapt G2-Si Mass spectrometer directly coupled to the chromatographic system. Protein expression was evaluated via a label-free ion mobility–enhanced data-independent acquisition (DIA) proteomics analysis in expression configuration mode (HDMSE). Processing of low and elevated energy, added to the data of the reference lock mass [Glu1]-Fibrinopeptide B Standard (Waters Corp.), provided a time-aligned inventory of accurate mass-retention time components for both the low- and the elevated-energy exact mass retention time (EMRT). Protein quantification was performed using PLGS 3.03 (Waters Ltd., Newcastle upon Tyne). The samples were automatically aligned according to retention time. The peak processing method was performed in profile data mode and the peptide ion detection method was set in high-resolution mode. Database search was performed interrogating the UniProt *B. taurus* reference proteome (proteome ID: UP000009136, 37513 protein entries, last release 21/09/2019). Peptide mass tolerance was set to 10 ppm and fragment ion tolerance was set to 0.01 Da. Carbamidomethyl cysteine and oxidation of methionine were selected as fixed and variable modifications, respectively. The search results were filtered to obtain a protein false discovery rate (FDR) of 1%.

#### *In vivo* Study

##### Animals

Forty-eight Holstein Friesian cows enrolled for this study were selected in two privately owned farms. In both farms, the animals were divided into groups based on the lactation period and were fed with a total mixed ration ad lib. Herd size varied from 150 to 200 cows in milk.

The farmers and the veterinarian enrolled in this study identified milk alterations at milking time. After a detailed clinical examination, the eligibility of the affected animal for the study was assessed. Cows requiring systemic antibiotic treatment were not enrolled in this study. Mastitis was classified as acute or chronic based on clinical signs and milk analysis. Acute mastitis was defined by the presence of abnormal milk (flakes and clots) and clinical signs such as hot, swollen, and painful quarter. In acute mastitis, milk production usually decreases and milk composition is considerably altered (40).

In this study, chronic mastitis was diagnosed when the somatic cell count (SCC) in the milk sample from the affected mammary quarter was >500,000 for three consecutive months (41).

The animals were randomly allocated to four groups:

– Acute mastitis that was treated with intramammary antibiotic (control group, CTR) (16 quarters/16 animals)
– Acute mastitis that was treated with intramammary CM (16 quarters/16 animals)
– Chronic mastitis that was treated with intramammary antibiotic (CTR) (8 quarters/8 animals)
– Chronic mastitis that was treated with intramammary CM (8 quarters/8 animals)

Each udder quarter affected by mastitis was enrolled only once.

##### Indicators of udder inflammation

*Somatic cells count*. Somatic cell count was evaluated at different time points: day 0, 7, 14, and 30 of treatment. The count was performed with a DeLaval Cell counter DCC (Tumba, Sweden) that performs fluorometric measurements. The somatic cell count is expressed as a linear score (LS), which is a logarithmic function that transforms the values of somatic cells into a linear scale:

$$\begin{array}{l} Linear\;Score=log_{2}(n^{{}^{\circ}}cells:100)+3 \end{array}$$

*Bacteriological analysis*. The bacteriological assays were performed on quarter milk samples collected before milking at the start of the treatment (day 0) and at day 30 by an aseptic procedure. Bacteriological analysis was performed by plating milk on blood agar with esculine and Baird Parker RPF Agar. The plates were incubated at 37°C for 48 h, and the colonies were identified by routine methods according to the National Mastitis Council (42).

##### Intramammary administration of antibiotics or CM

All cows were treated after milking. CM was warmed to 38.5°C and inoculated intramammarily through the teat canal with a syringe connected to a sterile disposable teat cannula. In the CTR group, the antibiotic for local use, chosen on an antibiogram test, was inoculated using the same procedure. Following inoculation, the udder was gently rubbed with upwards movement of the hands to distribute the product.

Three milliliters of CM alone or antibiotic alone was administered twice daily, after milking, for three consecutive days. The standard experimental volume of CM was set at 3 ml since, after lyophilization, CM was concentrated 4-fold, and a uniform treatment protocol was needed to compare to the antibiotic treatment.

After these treatments, an iodine teat-dip was performed.

##### *In vivo* results

An expert veterinarian defined clinical improvement when the quarter and milk secretion subjectively returned to normal, and relapse was defined as a clinical mastitis that re-occurred from the time of last examination (30 days after the treatment) until the term of the same lactation. A quarter was considered clinically recovered when clinical improvement occurred and an SCC value at T30 reduced by at least 3 LS points compared to the value at T0.

### Statistical Analysis

Data were analyzed by GraphPad Instat 3.00 for Windows (GraphPad Software, La Jolla, CA, USA).

For *in vitro* study, all experiments were replicated three times, and the data of viable, apoptotic, and necrotic epithelial mammary cells, collected in different experimental conditions, were expressed as percentages. Mean values and SEM of three replications were compared using ANOVA.

For *in vivo* study, the between-subjects factor was represented by treatments (2 levels) and the within-subjects factor was represented by sampling time (4 levels) and the mixed-design analysis of variance model was applied.

For all tests, differences were considered statistically significant at *p* ≤ 0.05.

The ClueGO plugin v 2.5.5 (release 15.10.2019) for Cytoscape 3.7.2 (43) was used to obtain the ontology classification and functional interaction networks for the proteins identified (44). Functions associated with the groups were partitioned based on significant functional associations between terms and protein sets. Gene ontology (GO) categories and pathways that included biological processes (BPs), molecular functions (MFs), and Kyoto Encyclopedia of Genes and Genomes (KEGG) updated at the last release (11.01.2018) were used. A two-sided hypergeometric test with Bonferroni correction was used to select only significative annotations with a *p* ≤ 0.05.

## Results

### *In vitro* Study

#### Cell Isolation and CM Production

Amniotic cells were chosen for their ability to adhere to plastic. Trypan blue exclusion assay showed an initial viability of AMCs >90% and >80% for BME-VU cells. As a characteristic of MSC phenotype, the AMCs at P3 express *CD29, CD44, CD73, CD106, CD105, Oct-4, c-Myc*, and *MHCI*, and lack *CD34* and *MHCII*. Moreover, AMCs are able to differentiate into mesenchymal (adipogenic, chondrogenic, and osteogenic differentiation) and ectodermic lineages (neurogenic differentiation) as reported by Corradetti et al. (34).

#### Cell Viability and Apoptosis

**Figure 1:**
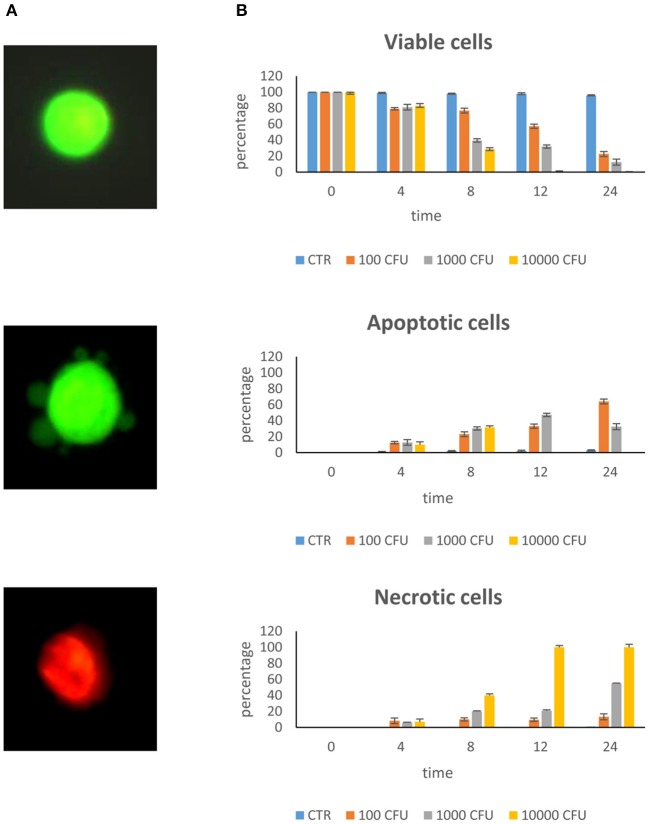
Dose–response curve. Epithelial mammary cells were cultured with *S. aureus* inoculated at the rate of 100, 1,000, and 10,000 CFU/ml for 4, 8, 12, and 24 h. **(A)** Example of combined AO/PI staining. Under fluorescence microscopy, a viable cell showed green preserved chromatin; a necrotic cell showed orange-red nuclei staining; an apoptotic cell showed green fragmented chromatin and budding of membrane. **(B)** Rate of viability, apoptosis, and necrosis.

Figure 1A shows the different cell lines after staining for viability. Viable cells appear with normal green-colored nuclei and well-defined margins; apoptotic cells show green, or orange chromatin and have membranes with buds; necrotic cells have orange or red chromatin.

#### Dose–Response Curve

The dose–response curve of *S. aureus* on BME-UV cells showed that maximum stress, as estimated by cell apoptosis measurements, occurred with 10,000 CFU/ml and 24 h incubation (Figure 1B). At this time, cells invaded by *S. aureus* detached from the culture plate and appeared as brown clusters in the culture media.

#### Treatment With *S. aureus* and CM

After evaluation of data obtained by the dose–response curve, all *in vitro* experiments were performed with BME-UV cells in the presence of 10,000 CFU/ml for 4, 8, 12, and 24 h at 38.5°C in 5% CO_2_.

**Figure 2:**
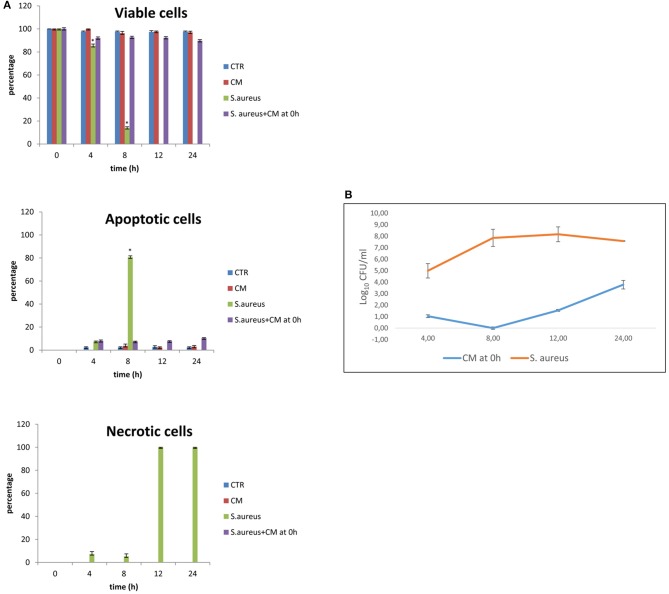
Co-culture of BME-UV cells and *Staphylococcus aureus* in the presence of 10% CM added at 0 h. **(A)** Rate of viability, apoptosis, and necrosis. **(B)** Number of CFUs in the presence of CM or *S. aureus* alone. Mean values with a star (*) are significantly different from the rest within the same time point (*P* < 0.05).

Results of the experiment of co-culture of cells and *S. aureus* in the presence of 10% CM added at time 0 are shown in Figures 2A,B for viability and apoptotic staining and for number of CFUs. These results demonstrate that the inoculation of *Staphylococcus* in the absence of CM induces cell death by 12 h. In the presence of 10% CM, 89.67% of cells remain viable for 24 h in culture. CM can attenuate bacterial growth; indeed, the bacterial counts remained at lowest levels until 24 h compared to the *S. aureus* alone.

**Figure 3:**
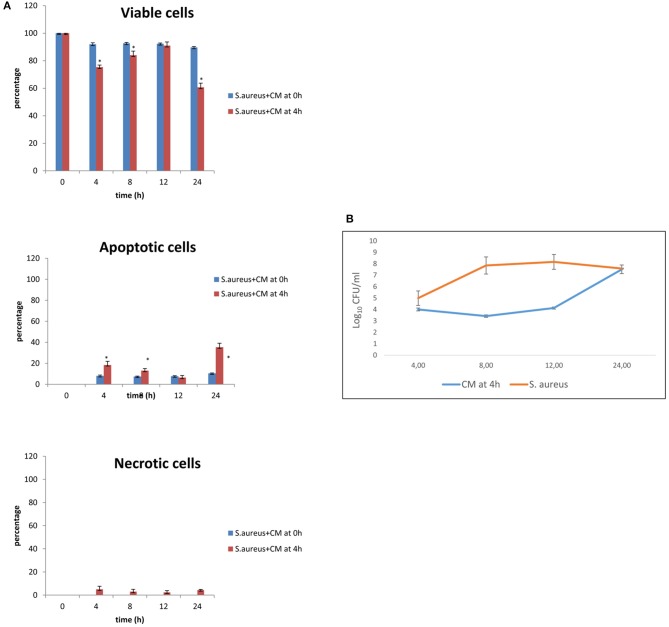
Co-culture of BME-UV cells and *S. aureus* in the presence of 10% CM added at 0 h or 4 h. **(A)** Rate of viability, apoptosis, and necrosis. **(B)** Number of CFUs in the presence of CM or *S. aureus* alone. Mean values with a star (*) are significantly different from the rest within the same time point (*P* < 0.05).

The results of co-culture of cells and *S. aureus* in the presence of CM added 4 h after bacterial inoculation are shown in Figures 3A,B for viability and apoptotic staining and for number of CFUs. These results demonstrate that, at 24 h, 60.67% of cells with 10% of CM remain viable. When CM was added 4 h after bacterial inoculation, bacterial counts increased to a level close to 10^8^ CFU/ml only at 24 h.

The effects of *S. aureus* and CM on basal cell viability, apoptosis, and necrosis were time-related and associated with the time of addition of CM (0 or 4 h after the inoculation of *S. aureus*). The same effects were observed for bacterial counts.

#### Protein Identification in CM

**Figure 4:**
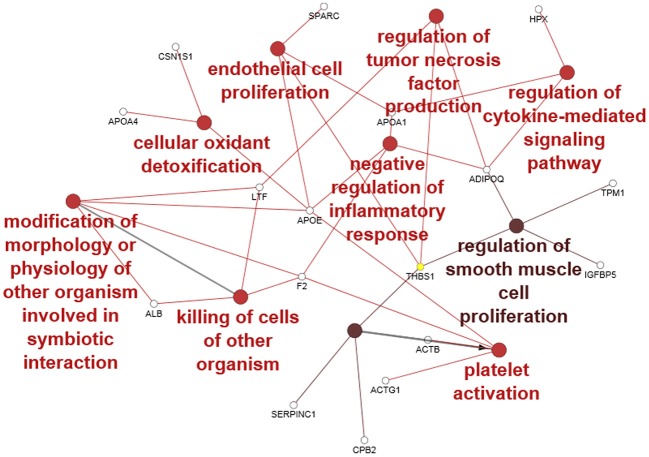
ClueGO cytoscape network of CM proteins associated with GO biological processes (GO BP) at *medium* network specificity. Colors of each circle represent the significance level (red pV 0.005–0.05, light brown pV 0.0005–0.005) and the radius of the circle denotes the number of mapped genes/proteins (0–5). Empty small circles represent the mapped proteins. Conversion Gene, protein respectively; LTF, lactoferrin; APOA4, apolipoprotein A4; F2, Prothrombin; ALB, albumin; APOE, apolipoprotein E; ACTB, actin B; ACTG1, actin G1; CBP2, Carboxypeptidase B2; CSN1S1, Alpha-S1-casein; THBS1, Thrombospondin-1; APOA1, apolipoprotein A1; ADIPOQ, Adiponectin; TPM1, Tropomyosin alpha-1 chain; IGFBP5, Insulin-like growth factor-binding protein 5; HPX, hemopexin; SERPINC1, Antithrombin-III.

Forty-seven *Bos taurus* proteins were identified in the CM (see Supplementary File 1). To obtain ontologies and functions for the proteins, the entire list was analyzed with the software ClueGO. Forty-three proteins were functionally annotated to all the ontologies available and 22 were associated with 35 representative terms and pathways after two-sided hypergeometric test with Bonferroni correction. To provide simple illustration of the most interesting terms and pathways associated with this list, a ClueGO network representation was produced (Figure 4). In this figure, we selected the most significant GO BP terms (red to brown circles) associated with several of the identified proteins (empty small circles). Eight GO BP terms belong to functions important in explaining the potential effect of CM during the *in vitro* and *in vivo* challenge.

### *In vivo* Results

#### Somatic Cells Count and Bacteriological Assay

Forty-eight animals were enrolled in this study: 32 affected by acute mastitis and 16 by chronic mastitis. The allocation of the enrolled quarters into the two different experimental groups was random. A list of the different species of bacteria identified is also provided (Tables 2, 3).

**Table 2:** Allocation of enrolled quarters with acute mastitis into two experimental groups and bacterial types cultured.

**Quarters**	**Total *n*** **(%)**	**Antibiotic** **(%)**	**CM** **(%)**
Total treated	32 (100)	16/32 (50)	16/32 (50)
Positive to bacterial growth	23 (71.88)	12/16 (75.00)	11/16 (68.75)
**Microorganism**			
Aesculin-positive *Streptococci*	8/23 (34.78)	4/8 (50)	4/8 (50)
Coagulase-negative *Staphylococci*	6/23 (26.09)	3/6 (50)	3/6 (50)
*Escherichia coli*	4/23 (17.39)	2/4 (50)	2/4 (50)
*Streptococcus dysgalactiae*	3/23 (6.79)	1/3 (25.00)	2/3 (75.00)
*S. aureus*	1/23 (4.35)	1 (100)	–
Others	1/23 (4.35)	1 (100)	–

**Table 3:** Allocation of enrolled quarters with chronic mastitis into two experimental groups and bacterial types cultures.

**Quarters**	**Total *n*** **(%)**	**Antibiotic** **(%)**	**CM** **(%)**
Total treated and positive for bacterial growth	16 (100)	8/16 (50)	8/16 (50)
**Microorganism**			
Aesculin-positive *Streptococci*	8 (50.00)	4/8 (50.00)	4/8 (50.00)
*S. aureus*	8 (50.00)	4/8 (50.00)	4/8 (50.00)

#### Treatments

Table 4 shows the rate of quarters with a reduction of at least 3 LS compared to the starting point and the rate of relapses.

**Table 4:** Somatic cells count values and rate of relapse for each treatment, and clinical distinction between acute and chronic mastitis.

**Mastitis**	**Treatment**	**N^**∘**^ treated** **quarters**	**N^**∘**^ quarters** **with >3 LS** **(%)**	**N^**∘**^ quarters with** **improvement** **<3 LS** **(%)**	**No. of quarters with improvement that relapsed <3 LS** **(%)**	**N^**∘**^ quarters with improvement < 3 LS without relapse** **(%)**
ACUTE	Antibiotic	16	7/16 (43.75)^a^	9/16 (56.25)^a^	6/9 (66.67)^a^	3/9 (33.33)^a^
	CM	16	8/16 (50.00)^a^	8/16 (50.00)^a^	0/8 (00.00)^b^	8/8 (100)^b^
CHRONIC	Antibiotic	8	8/8 (100.00)^A^	0/8 (0.00)^A^	0/8 (0.00)^A^	0/8 (0.00)^A^
	CM	8	6/8 (75.00)^A^	2/8^*^ (25.00)^A^	0/8 (0.00)^A^	2/8 (100.00)^A^

*Different small letter superscripts (a,b) in the same column indicate statistically different comparisons (p < 0.05) between acute mastitis*.

*Different capital letter superscripts (A,B) indicate statistically different comparisons (p < 0.05) between chronic mastitis*.

* *Two animals affected by chronic mastitis due to Staphylococcus aureus*.

There was no difference in the improvement of clinically affected quarters treated with CM compared to antibiotic treatment but the rate of relapses was numerically different.

Treatment with CM improved two quarters affected by chronic mastitis.

Table 5 shows the values of the somatic cells expressed in LS at the different time points for acute and chronic mastitis treated with antibiotics or CM. At time 0 (T0), the average value of SCC was 7.68 LS with a standard deviation (SD) of 0.30 in acute mastitis and 8.40 LS with a SD of 0.18 in chronic mastitis. There was no statistically significant difference between the antibiotic group compared to CM groups but, in the antibiotic treatment, the mean value of LS decreased constantly compared to the CM treatments. Indeed, in both acute and chronic mastitis, the reduction of LS values in the CM-treated group became evident at the 14th day from the beginning of treatment compared to the antibiotic alone in which the decrease started at the 7th day. The results of both treatments (CM and the antibiotic) were comparable.

**Table 5:** Mean values of the linear scores on days 0, 7, 14, and 30 in cows with acute and chronic mastitis treated with antibiotic or CM.

**Mastitis**	**Treatment**	**Day 0**	**Day 7**	**Day 14**	**Day 30**
Acute	Antibiotic	7.46 ± 1.33^aA^	6.83 ± 1.69^aA^	5.92 ± 3.12^aA^	4.37 ± 2.66^bA^
	CM	7.89 ± 1.11^aA^	7.63 ± 1.45^aA^	6.78 ± 1.28^aA^	4.75 ± 2.22^bA^
Chronic	Antibiotic	8.27 ± 0.45^aA^	6.48 ± 1.31^bA^	6.59 ± 1.03^bA^	6.28 ± 0.77^bA^
	CM	8.52 ± 1.12^aA^	7.98 ± 1.26^aA^	7.08 ± 1.21^aA^	6.11 ± 1.15^bA^

*Different small letter superscripts (a,b) in the same line indicate significantly different comparisons (p ≤ 0.05) within the same treatment at different days in acute or chronic mastitis. Different capital letter superscripts (A,B) in the same column indicate significantly different comparisons (p ≤ 0.05) among treatments in acute or chronic mastitis*.

## Discussion

In the present study, we hypothesized that the secretome produced by amniotic-derived cells might have anti-inflammatory roles that contribute to protection against infection by *S. aureus*. In this context, we investigated the effect of CM in an *in vitro* experiment on epithelial mammary cells infected by *S. aureus* and in an *in vivo* study on cows with acute and chronic mastitis. Mastitis is an inflammatory process of the mammary gland that results, not only in decreased milk production, but also in a reduction in milk quality. The action of pathogens, mainly bacteria, and the consequent inflammatory process induce a change in the integrity and function of the mammary epithelial cells with a resultant decrease in milk production (45, 46). Therapy for mastitis (acute, chronic) and its prevention (for dry cows) is mainly based on the use of antibiotics but, in recent years, antibiotic resistance has been developing to *S. aureus*, which is the most prevalent and contagious etiologic agent in mastitis (47). Infections caused by these bacteria are difficult to treat, mainly due to evolved mechanisms of antimicrobial drug resistance and evasion of immune response (48–50). Because of the growing number of antibiotic-resistant *S. aureus* strains, there is an urgent need to investigate and develop alternative therapeutics.

Bovine mammary epithelial cells constitute a physical barrier and produce several antimicrobial substances and inflammatory mediators such as tumor necrosis factor-alpha (TNF-α), interleukin-1 beta (IL-1β), granulocyte macrophage colony-stimulating factor (GM-CSF), interleukin-8 (IL-8), regulated on activation normal T cell expressed and secreted (RANTES), lactoferrin, serum amyloid A, and cyclooxygenase-2 (46, 51–53). When severe, mastitis induces a decrease of α-lactalbumin and casein mRNAs and an increase in mRNA levels for several growth factors previously cited. These results suggest the involvement of growth factors in tissue protection or repair processes (54). It seems that the mammary gland tries to regenerate itself and, in this context, the use of CM which is rich in growth factors, could help the regenerative process (as described in literature for other injured tissues) (55–58). To date, however, there are few documented reports of the antimicrobial properties of CM (19) and so, in this study, this was investigated in *in vitro* and *in vivo* studies.

Our results show that CM is able to attenuate the proliferation of *S. aureus* in BME-VU cells either when added at time 0 or, to a lesser extent, when supplemented 4 h after the cells were exposed to this bacterium. In the presence of *S. aureus*, all BME-VU cells die within 12 h but when CM is added to the cell culture at the same time as the bacteria or 4 h later, 89.67 or 60.67% of cells, respectively, remain viable.

To confirm the ability of CM to counteract the proliferation of *S. aureus* and to verify its regenerative effect, an *in vivo* study was performed.

*In vivo*, the clinical diagnosis of mastitis is based on the physical examination of milk and udder and, above all, according to the recommendations of the International Dairy Federation (IDF), on SCC and on the microbiological status of the mammary quarter (59).

A positive bacterial culture was detected in 71.88% of animals with acute mastitis, while bacterial culture was positive in all cows with chronic mastitis.

Our results show that SCC values, expressed as LS, decreased significantly (*p* ≤ 0.05) at 30 days of observation (T30) in both mastitis groups, and no statistically significant differences were observed between antibiotic and CM-treated quarters. However, the CM group had a lower rate of relapses. Indeed, mastitis did not recur in any of the quarters after CM treatment.

The reduced recurrence rate in the CM group compared to the antibiotic group and the improvement of the quarters affected by chronic mastitis could be explained by the presence of growth factors in the CM with both regenerative and antibacterial effects. In fact, the number of quarters with positive evolution was higher in the CM-treated group compared to the antibiotic one.

Our proteomic study identified some BP terms associated with several identified proteins: in particular, the “negative regulation of inflammatory response” and “regulation of TNF production” could contribute via the action of the associated protein adiponectin (ADIPOQ), thrombospondin-1 (THBS1), lactoferrin (LTF), apolipoprotein E (APOE), apolipoprotein A1 (APOA1), and prothrombin (F2) to decrease TNF production and inflammation during the challenge with *S. aureus*. The presence of the term “killing of cells of other organism” associated with LTF, albumin (ALB), and F2 proteins suggests an antibacterial effect mediated mainly by F2 and LTF. The “endothelial cell proliferation” as well as “regulation of smooth muscle cell proliferation” may support a potential regenerative effect at the cellular level by associated proteins thrombospondin-1 (THBS1), insulin-like growth factor-binding protein 5 (IGFBP5), ADIPOQ, tropomyosin alpha-1 chain (TPM1) APOE, APOA1, and secreted protein acidic and cysteine rich (SPARC). Oxidant detoxification and related proteins could contribute to a decrease in oxidative stress and indirectly improve cell viability. The term platelet activation may be related to the release of proteins into the CM during growth. Taken together, all the functions and the related proteins associated with the CM support an anti-inflammatory, anti-bacterial, and proliferative effect of CM.

Many studies have confirmed the antimicrobial effects of CM. Studies using models of infection-induced organ damage have shown that MSCs have direct and indirect antimicrobial properties through the release of soluble factors, such as the peptide catelicidine LL-37 (20), lipocalin-2 (19, 21) and beta-defense-2 (60), which lead to bacterial clearance.

Other anti-bacterial mechanisms of MSCs could include tryptophan catabolism through indoleamine 2,3-dioxygenase (61) or through increased phagocytosis (57–60). MSCs increase macrophage phagocytosis of bacteria by inducing the phenotypic transition of monocytes from type 1 to type 2 (21, 62–67). In a mouse model with peritonitis induced by *Pseudomonas aeruginosa*, MSCs reduced the number of colony-forming units in the blood by increasing the phagocytic activity of monocytes (66). In a mouse model of sepsis (ligation and perforation of the caecum), bacterial colonies in the blood decreased in the group of animals treated with MSCs. The authors speculate that this effect can be explained by the call of neutrophils within the vascular compartment mediated by IL-10 (68). Equine MSCs from different tissue are able to express some immunomodulatory genes such as monocyte chemoattractant protein-1 (*MCP-1*), interleukin-6 (*IL-6*), interleukin-8 (*IL-8*), and chemokine ligand-5 (*CCL5*), suggesting that respective cytokines may help limit infection indirectly by recruiting and activating immune cells (19).

The action of these growth factors, which attract neutrophils and macrophages, could help explain why the SCC remained high throughout CM treatment in our study, decreasing only from T14 and not at T7 as in the antibiotic treatment.

In summary, we suggest that CM contains growth factors with tissue regenerative and antimicrobial action, as well as chemokines, which could act, in synergy, to increase the host non-specific immune mechanism, promoting angiogenesis, fibroplasia, matrix deposition, and re-epithelialization.

## Conclusion

The use of CM for the treatment of mastitis would minimize the use of antibiotics, limiting antibiotic resistance, and avoiding ineffective and expensive treatments. Moreover, chronic mastitis cases are often not treated, and affected animals are culled from the herd. If our preliminary findings are confirmed in a larger study, it may be possible to restore milk production even in cows that would normally be destined to leave the production cycle. The use of CM may have further economic advantages for the effective recovery of the glandular tissue and most importantly for the prevention of antibiotic residues in the milk. In our *in vitro* experiment, secretome produced by amniotic-derived cells had protective effects on epithelial mammary cells infected by *S. aureus*. Finally, the use of CM avoids the risk of rejection compared to the transplantation of original MSCs (69), but further studies will be needed to fully clarify antimicrobial effects of CM.

## Data Availability Statement

All datasets generated for this study are included in the article/Supplementary Material.

## Ethics Statement

The study was approved by the University of Milan Ethics Committee (Protocol No. 119-2017). All procedures were conducted following standard veterinary practice and in accordance with 2010/63 EU directive on animal protection. The informed client consent was obtained for collection placenta at term of cow pregnancies and for cow treatments.

## Author Contributions

AL-C: conception and design, isolation of amniotic cells and production of conditioned medium, mammary cell culture, coordination of all experiments, collection and assembly of all data, analysis and interpretation of data, manuscript writing, and final approval of manuscript. CG: *in vitro* experiment with *Staphylococcus aureus*, collection and assembly of data of CFU, bacteriological analysis, SCC, interpretation of data, and final approval of manuscript. EM: *in vivo* study with collection of sample for bacteriological analysis and SCC, *in vivo* administration of PRP, evaluation of outcomes, and final approval of manuscript. AI: cell cultures, cell staining, collection and assembly of data of vitality, and final approval of manuscript. AS, LB, and VG: proteomic analysis, interpretation of data, financial support, and final approval of manuscript. FC: conception and design, *in vivo* study with administration of PRP, evaluation of outcomes, interpretation of data, financial support, and final approval of manuscript. AZ: conception and design, analysis and interpretation of data, financial support, and final approval of manuscript.

### Conflict of Interest

The authors declare that the research was conducted in the absence of any commercial or financial relationships that could be construed as a potential conflict of interest.

## Supplementary Material

Supplementary File 1 Forty-seven bos taurus proteins identified in the conditioned medium.

## Abbreviations

ACTB: actin B
ACTG1: actin G1
ALB: albumin
ADIPOQ: adiponectin
AO: acridine orange
APOA1: apolipoprotein A1
APOA4: apolipoprotein A4
APOE: apolipoprotein E
BME-UV cells: bovine epithelial mammary cells
CBP2: carboxypeptidase B2
CCL5: chemokine ligand-5
CFU: colony-forming units
CM: conditioned medium
CSN1S1: alpha-S1-casein
CTR: control group
FBS: inactivated fetal bovine serum
F2: prothrombin
GM-CSF: granulocyte macrophage colony-stimulating factor
HG-DMEM: high-glucose Dulbecco's modified Eagle's medium
HPX: hemopexin
IGFBP5: insulin-like growth factor-binding protein 5
IL-1β: interleukin-1 beta
IL-6: interleukin-6
IL-8: interleukin-8
LPS: lipopolysaccharide
LS: linear score
LTF: lactoferrin
MCP-1: monocyte chemoattractant protein-1
MSCs: mesenchymal stromal cells
PI: propidium iodide
RPMI: Roswell Park Memorial Institute
*S. aureus*: *Staphylococcus aureus*
SCC: somatic cells count
SD: standard deviation
SERPINC1: antithrombin-III
THBS1: thrombospondin-1
SPARC: secreted protein acidic and cysteine rich
TNF-α: tumor necrosis factor-alpha
TPM1: tropomyosin alpha-1 chain.
